# HOW AGE-FRIENDLY UNIVERSITIES CAN COMBAT AGEISM

**DOI:** 10.1093/geroni/igac059.1408

**Published:** 2022-12-20

**Authors:** Allyson Graf, Heather Han, Will Phillips, Jessica Wiley

**Affiliations:** Northern Kentucky University, Florence, Kentucky, United States; Northern Kentucky University, Highland Heights, Kentucky, United States; Northern Kentucky University, Highland Heights, Kentucky, United States; Northern Kentucky University, Highland Heights, Kentucky, United States

## Abstract

At a time of growing enrollment among adult learners, student bodies are increasingly age diverse. Identifying and reducing age-related bias on campus may be multipurpose in supporting those who experience ageism while promoting professional skills to combat ageism in students' future careers. The extant literature has primarily focused on ageism in the workplace and ageism experienced by faculty and staff in higher education while less has focused on ageism experienced by adult students and the possible disruption this creates to feeling included as part of the campus community. We present data from a mixed methods study of adult learners assessing the extent to which they experience and are impacted by age-related bias on campus. Data are used to justify faculty and student training to improve intergenerational contact in the classroom and beyond under the broader umbrella of DEI initiatives.

